# Supplementary material for: Computationally profiling peptide:MHC recognition by T-cell receptors and T-cell receptor-mimetic antibodies
Source: Front Immunol. 2023 Jan 9;13:1080596. doi: 10.3389/fimmu.2022.1080596 (PMC9868621; doi:10.3389/fimmu.2022.1080596)
Supplement: Supplementary file 1 [file DataSheet_1.pdf]

Supplementary Material for ‘Computationally profiling peptide:MHC recognition by T-cell receptors and T-cell receptor-mimetic antibodies’

SUPPLEMENTARY METHODS

TCR:pMHC and TCRm:pMHC structure preparation for molecular dynamics

X-ray crystal structures of four pMHC-TCRm and eight pMHC-TCR complexes were obtained from the Protein Data Bank (Berman et al., 2000). Missing loops and heavy atoms were rebuilt using CHARMM35 (Brooks et al., 2009) based on SEQRES records and default residue parameters (see Supplementary Table 3); short sections of missing residues at N- and C-termini were not rebuilt. Despite the increased simulation cost, TCR and TCR-mimetic antibody (TCRm) constant regions were included as this was recommended by previous studies (Knapp et al., 2017). The H++ webserver (Anandakrishnan et al., 2012) (http://biophysics.cs.vt.edu/H++) was used to determine protonation states for all titratable groups in these rebuilt structures assuming a pH of 7 and internal and external dielectric constants of 10 and 80, respectively, at a salt concentration of 0.15 M. Amber-format parameter/topology and coordinate files were prepared for periodic boundary simulations with tleap (Salomon-Ferrer et al., 2013) using FF14SB (Maier et al., 2015) and TIP3P (Jorgensen et al., 1983) forcefield parameters for protein and water, respectively. The PBRadii setting mbondi2 was used to prepare all solvated and dry topology files. Each complex was solvated in an orthorhombic box with a minimum distance between protein atoms and the box edge of 1.4 nm. Sodium and chloride ions were then added to neutralize each system and bring the salt concentration to 0.15 M, with ion counts chosen using the SPLIT method (Machado and Pantano, 2020).

Molecular dynamics simulation preparation

All minimization, heating, and production simulations were carried out using OpenMM v7.5 (Eastman et al., 2017). In all simulations, bonds containing hydrogen were constrained, the particle-mesh Ewald method (Darden et al., 1993) was used to compute long-range electrostatic interactions, and a non-bonded cutoff of 1 nm was used to compute short-range non-bonded interactions. All NVT simulations were conducted with a Langevin Middle Integrator (Zhang et al., 2019) with a friction coefficient of 1 ps^{-1} and integration time step of 2 fs. NPT simulations were conducted identically to NVT simulations but with the addition of a Monte Carlo barostat (updated every 10 integration time steps) to maintain system pressure at 1 bar. The rebuilt, solvated, and neutralized structures were first minimized for 1,000 iterations with spherical harmonic restraints on all protein heavy atoms with force constants of 10 kcal/[mol x $Å^2$]. Systems were then heated from 48 to 298 K in steps of 10 K in the NVT ensemble for 10 ps at each temperature with all protein heavy atom restraints maintained. After heating, another round of 1,000 iterations of minimization was performed, with spherical harmonic restraints with force constant 5 kcal/[mol x $Å^2$] on all protein C_α atoms. A second round of heating was then carried out, again in the NVT ensemble, from 48 to 298 K in steps of 10 K with C_α atom restraints maintained and 2.5 ps of dynamics simulated at each temperature. Spherical harmonic restraints were then sequentially relaxed and the system density allowed to equilibrate during a series of 100-ps NPT simulations at 298 K with C_α atom restraint force constants of 5, 4, 3, 2, 1, 0 kcal/[mol x $Å^2$] with the final 100 ps of equilibration unrestrained.

Production simulations, MMGBSA calculations, and contact analysis

Production trajectories of 5-ns duration in the NPT ensemble were initiated from the final coordinates of the unrestrained equilibration. A total of thirty statistically independent trajectories were each run through this minimization, heating, equilibration, and simulation procedure for each pMHC-TCR or pMHC-TCRm system. A total of 3,000 simulation frames collected at 40-ps intervals within the final 4 ns of each 5-ns production trajectory (i.e., 100 frames from each of 30 trajectories per complex) were analyzed using the MMPBSA.py (Miller et al., 2012) program from AmberTools21 (Salomon-Ferrer et al., 2013). Complex, receptor, and ligand trajectories were all collected from simulations of the bound complex conducted as described above. Generalized-Born implicit solvent (Onufriev and Case, 2019) calculations were carried out with igb 2 (compatible with mbondi2) and saltcon set to 0.15. In all calculations the receptor was defined as the MHC Class I and beta-2-microglobulin chains plus the peptide antigen, while the ligand was defined as the two TCR or TCRm chains. MMGBSA energies were decomposed over all residues using the MMPBSA.py DECOMP functionality with idecomp set to 1. The fraction of the DECOMP energy, f_{DECOMP}, contributed by the peptide, MHC alpha 1 ($\alpha 1$) helix, or MHC alpha 2 ($\alpha 2$) helix was computed as:

$$f^{pep}_{DECOMP} = \frac{\sum^{pep} g(i)}{\sum^{pep} g(i) + \sum^{\alpha 1} g(i) + \sum^{\alpha 2} g(i)}, \tag{S1}$$

$$f^{\alpha 1}_{DECOMP} = \frac{\sum^{\alpha 1} g(i)}{\sum^{pep} g(i) + \sum^{\alpha 1} g(i) + \sum^{\alpha 2} g(i)}, \tag{S2}$$

$$f^{\alpha 2}_{DECOMP} = \frac{\sum^{\alpha 2} g(i)}{\sum^{pep} g(i) + \sum^{\alpha 1} g(i) + \sum^{\alpha 2} g(i)}, \tag{S3}$$

respectively. In these equations, $g(i)$ is the MMGBSA free energy change for residue i, and $\sum^{pep} g(i)$, $\sum^{\alpha 1} g(i)$, and $\sum^{\alpha 2} g(i)$ are summations over the per-residue free energy changes for all residues in the peptide antigen, $\alpha 1$ helix, and $\alpha 2$ helix, respectively. Fractional contributions from the CDRs of a TCR or TCRm were calculated in an analogous fashion, with the numerator a summation of the $g(i)$ values for a particular CDR and the denominator replaced by the sums of the energies over all six CDRs in the complex.

Measuring the tilt of the R65 guanidino group relative to the MHCα_1 helix

Two planes were defined corresponding to (i) the plane of the guanidino group in the R65 sidechain and (ii) the plane of the α_1 helix. The guanidino group plane was defined as the plane containing the three nitrogen atoms NH1, NH2, and NE (using Amber naming conventions). The plane of the α_1 helix was defined as the plane passing through the Cα atoms of residues 64, 66, and 68 (see Supplementary Fig. 7A). The angle between the two planes was computed using cpptraj as the dot product of their normal vectors for each of the 3,000 simulation frames collected for each system by ensemble sampling.

SUPPLEMENTARY FIGURES AND TABLES

The following pages contain eight supplementary figures and six supplementary tables.

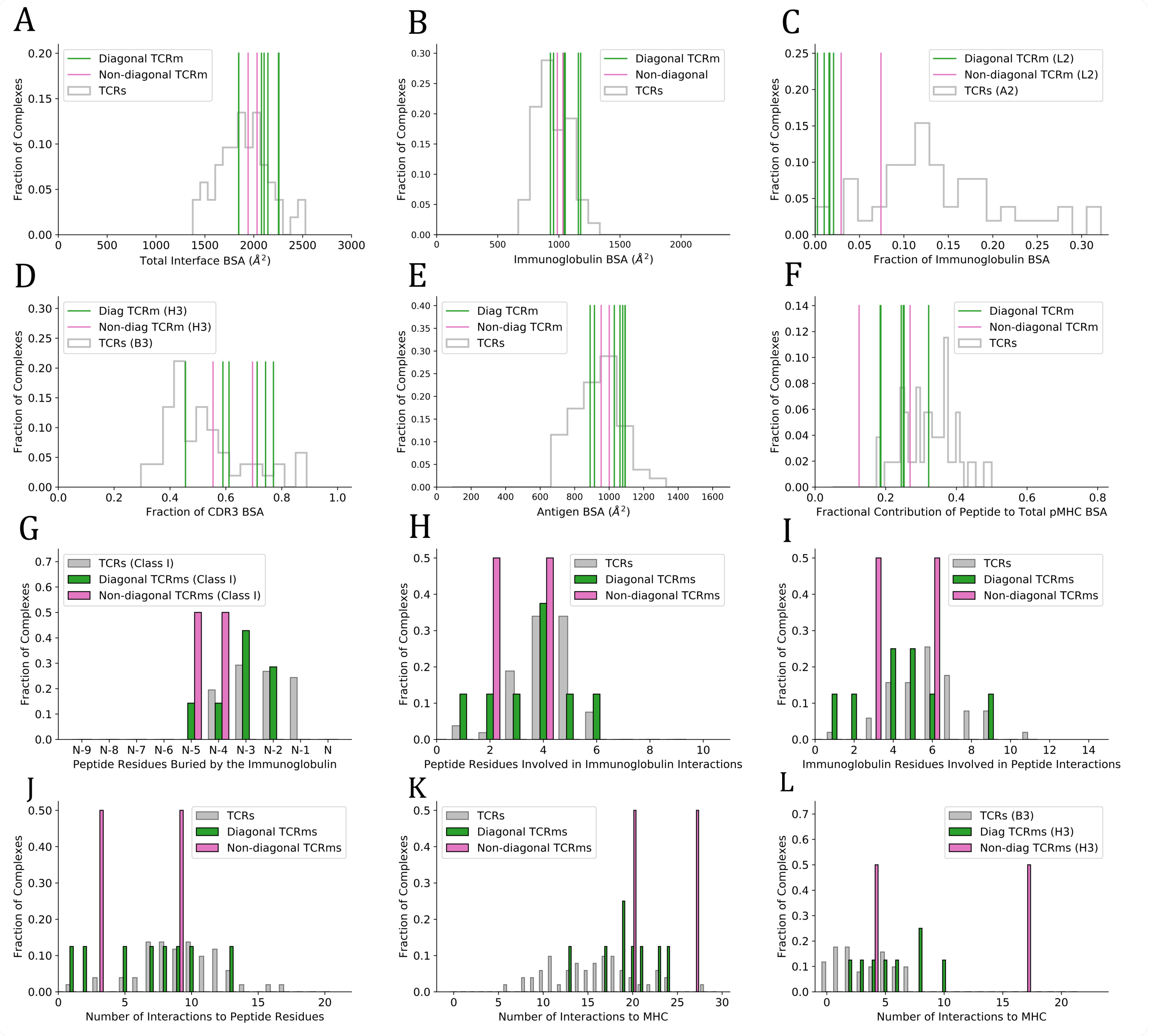

Figure S1. pMHC engagement properties of diagonal (green lines/bars) and non-diagonal (pink lines/bars) TCRms, in the context of the properties of representative TCRs (grey histograms). A: The total BSA across the immunoglobulin:antigen interface. B: The immunoglobulin portion of the total interface BSA. C: The fractional contribution of CDRL2 (TCRms) or CDRA2 (TCRs) to immunoglobulin BSA. D: The fractional contribution of CDRH3 (TCRms) or CDRB3 (TCRs) to CDR3 BSA. E: The pMHC portion of the total interface BSA. F: The fractional contribution of peptide BSA to pMHC BSA. G: The number of peptide residues buried in each immunoglobulin:pMHC Class I complex, expressed in terms of the total length of the peptide, N. The peptides range from N = 8 to N = 10 residues in length. H: The number of peptide residues involved in binding interactions to the TCRms/TCRs. I: The number of immunoglobulin residues involved in binding interactions to the peptide across the TCRms/TCRs. J: The number of interactions between the immunoglobulin and the peptide across the TCRms/TCRs. K: The number of interactions to MHC residues across the TCRms/TCRs. L: The number of interactions to MHC residues made by the CDRH3 (TCRms) or CDRB3 (TCRs) loop.

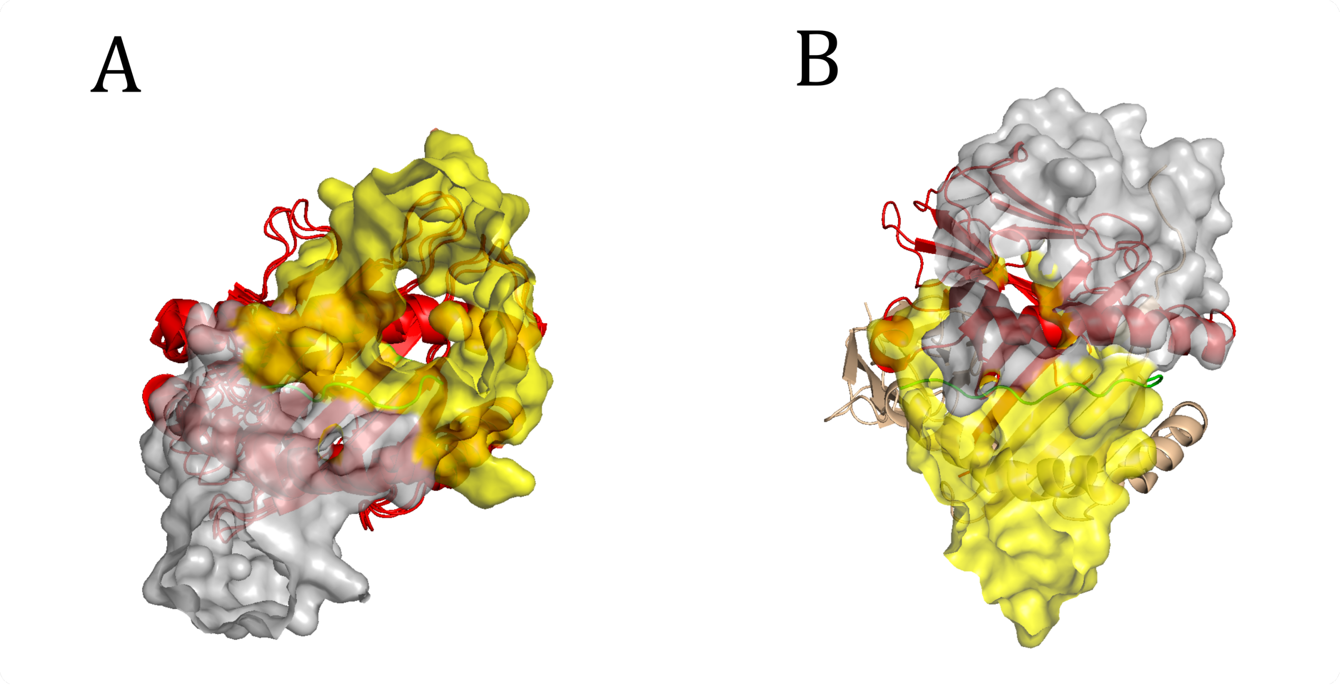

Figure S2. TCR diagonal binding modes to peptide:MHC. A: the canonical diagonal TCR:pMHC binding mode (here illustrated by 5WLG in the context of a class I pMHC), B: the ‘reverse polarity’ diagonal TCR:pMHC binding mode (here illustrated by 4Y19 in the context of a class II pMHC). Both exhibit similar absolute docking angles but with the roles of the TCR beta (VB) and alpha (VA) chains reversed. The non-canonical mode (B) is linked with weak/no T-cell signalling (Zareie et al., 2021). Red cartoon: MHC alpha chain; wheat cartoon: MHC beta chain; green cartoon: peptide; yellow surface: IMGT-defined VB (Lefranc et al., 2003), light gray surface: IMGT-defined VA (Lefranc et al., 2003).

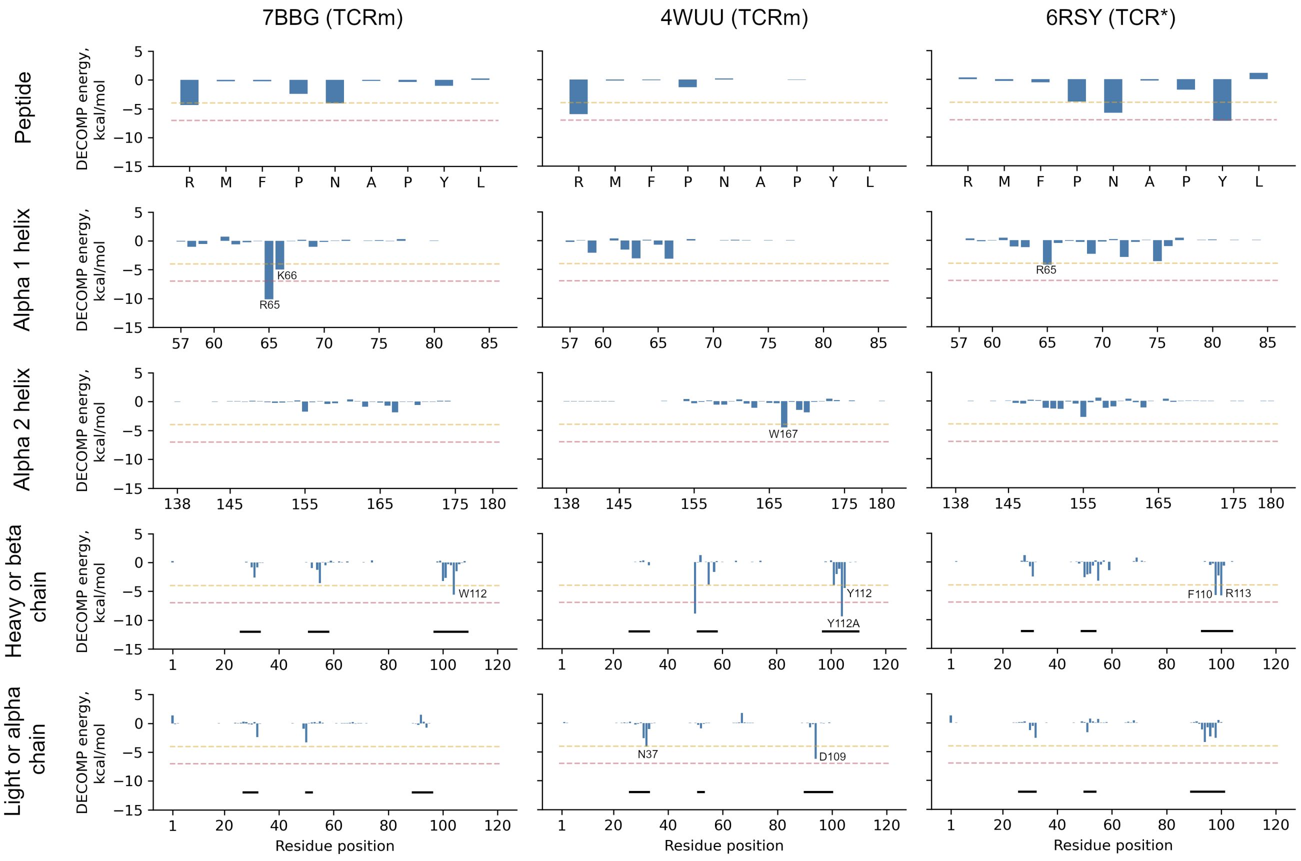

Figure S3. MMGBSA decomposition results (Miller et al., 2012) for all Wilms Tumor 1 pMHC-TCRm/TCR complexes. For TCR or TCR mimetic chains, x-axis labels give residue positions numbered sequentially from 1 while labels on individual peaks refer to CDR loop numbers generated with ANARCI (Dunbar and Deane, 2015) using the IMGT (Lefranc et al., 2003) scheme. Horizontal black bars indicate the locations of, from left to right, CDR1, CDR2, and CDR3 within the sequence. The dashed amber and red lines reflect the energy thresholds to be considered ‘semi-hotspots’ (-4 kcal/mol) and ‘hotspots’ (-7 kcal/mol), respectively. *Affinity-enhanced TCR. PDB code to immunoglobulin name mappings — 4WUU: ESK1, 6RSY: a7b2, 7BBG: 11D06.

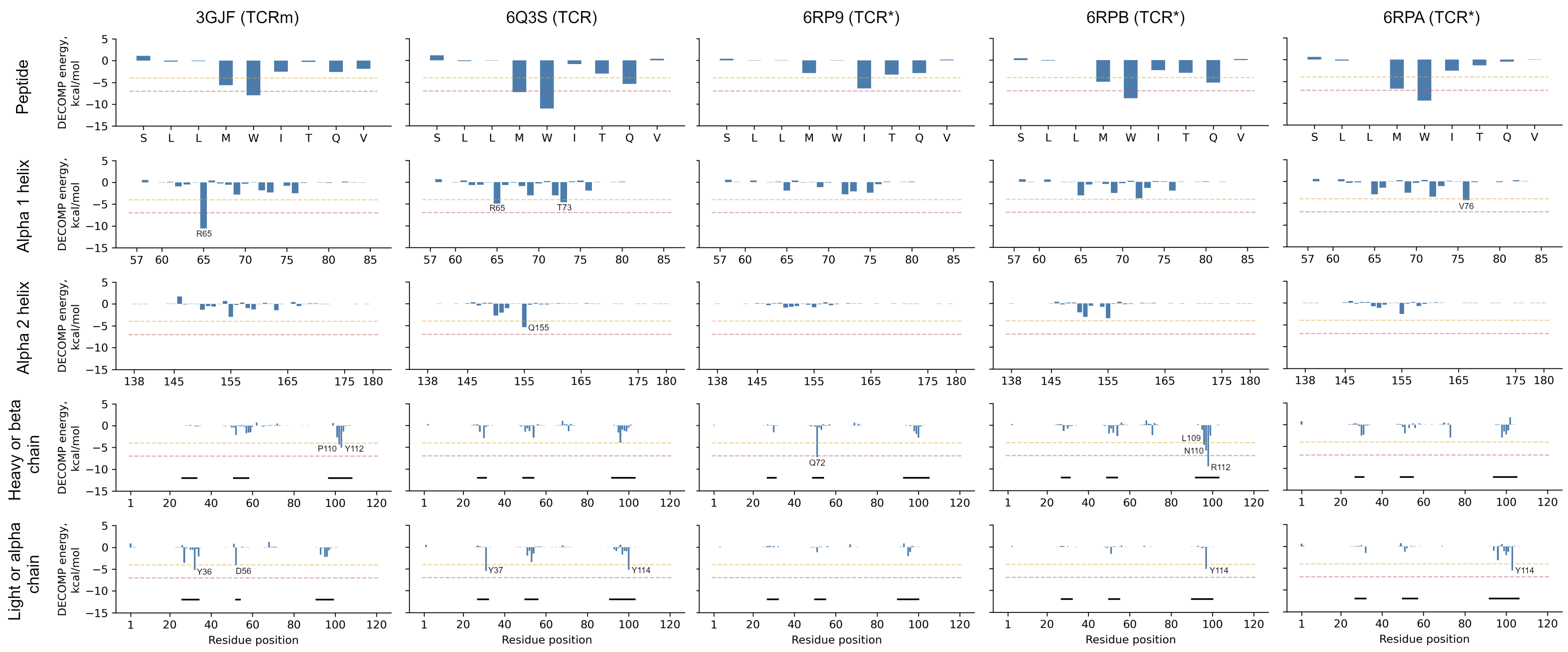

Figure S4. MMGBSA decomposition results (Miller et al., 2012) for all New York esophageal squamous cell carcinoma 1 (NY-ESO-1) pMHC-TCRm/TCR complexes. For TCR or TCR mimetic chains, x-axis labels give residue positions numbered sequentially from 1 while labels on individual peaks refer to CDR loop numbers generated with ANARCI (Dunbar and Deane, 2015) using the IMGT scheme (Lefranc et al., 2003). Horizontal black bars indicate the locations of, from left to right, CDR1, CDR2, and CDR3 within the sequence. The dashed amber and red lines reflect the energy thresholds to be considered 'semi-hotspots' (-4 kcal/mol) and 'hotspots' (-7 kcal/mol), respectively. *Affinity-enhanced TCR. PDB code to immunoglobulin name mappings — 3GJF: 3M4E5, 6Q3S: sp3.4, 6RPA: NYE_S2, 6RPB: NYE_S1, 6RP9: NYE_S3.

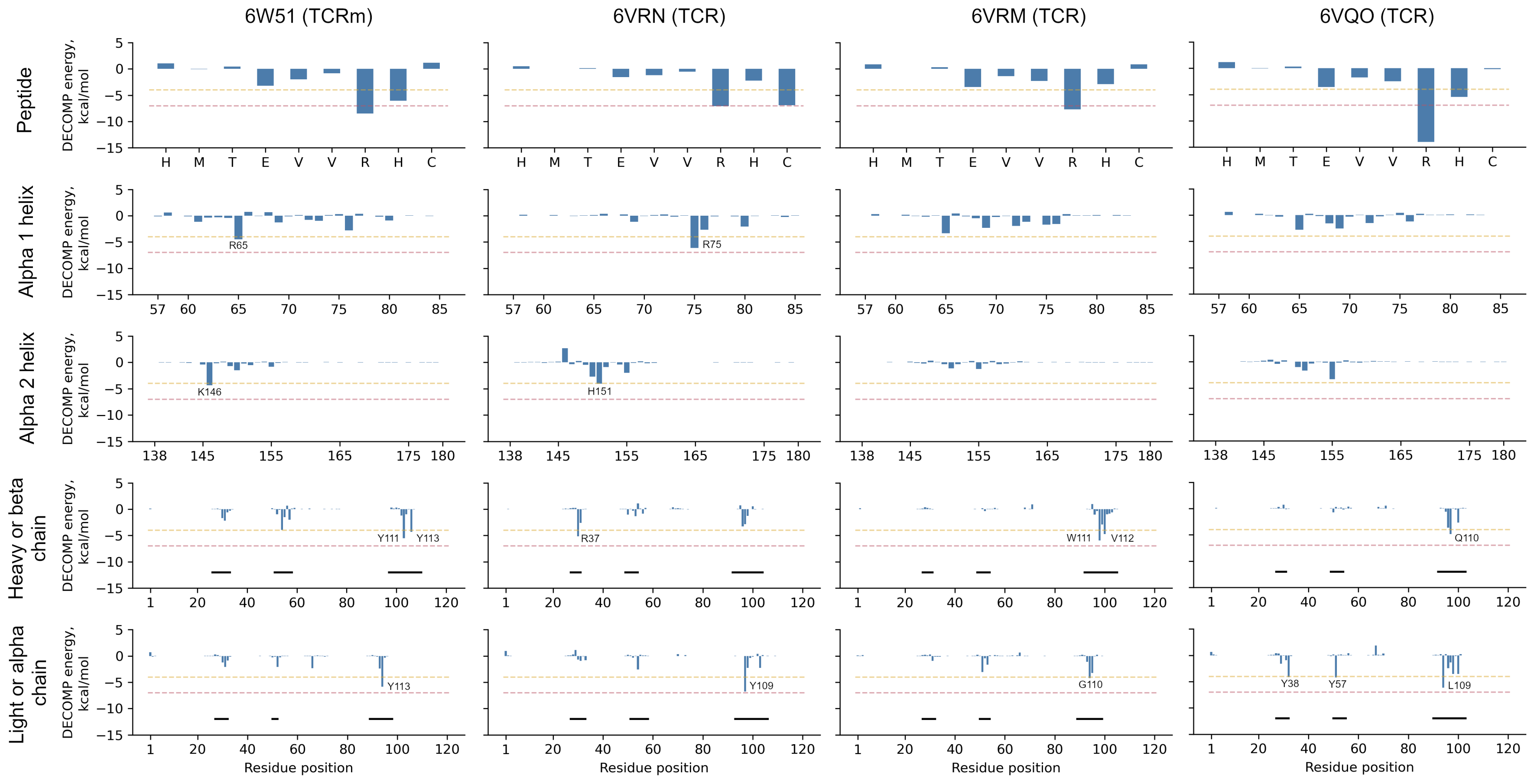

Figure S5. MMGBSA decomposition results (Miller et al., 2012) for all p53 R175H neoantigen (p53_R175H) pMHC-TCRm/TCR complexes. For TCR or TCR mimetic chains, x-axis labels give residue positions numbered sequentially from 1 while labels on individual peaks refer to CDR loop numbers generated with ANARCI (Dunbar and Deane, 2015) using the IMGT scheme (Lefranc et al., 2003). Horizontal black bars indicate the locations of, from left to right, CDR1, CDR2, and CDR3 within the sequence. The dashed amber and red lines reflect the energy thresholds to be considered 'semi-hotspots' (-4 kcal/mol) and 'hotspots' (-7 kcal/mol), respectively. PDB code to immunoglobulin name mappings — 6VQO: 1a2, 6VRM: 12-6, 6VRN: 38-10, 6W51: H2.

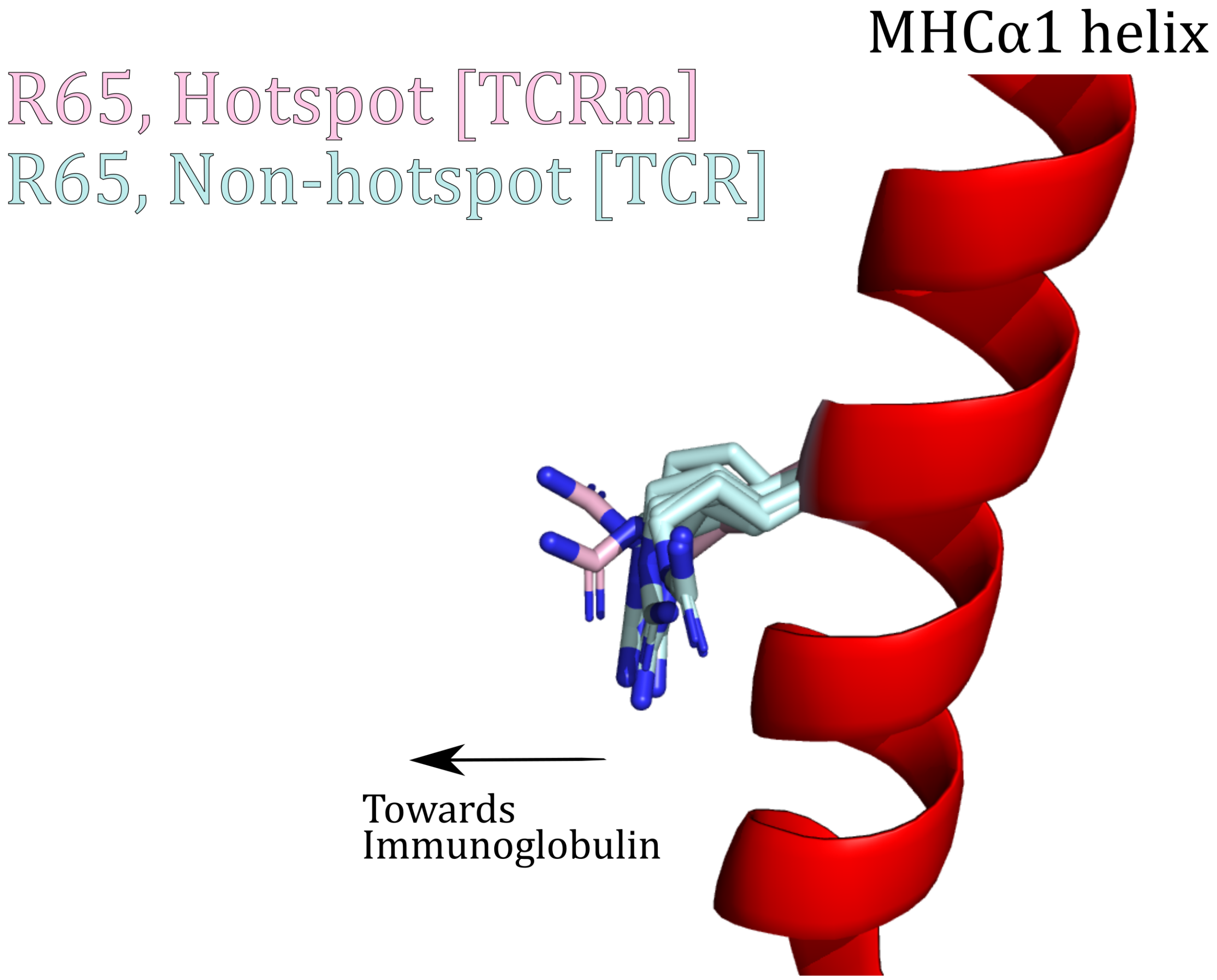

Figure S6. Using crystal coordinates, an alignment of the HLA-A2 MHCα_1 chain of TCRms with R65 energetic hotspots [3GJF, 7BBG; light pink] and TCRs without R65 energetic hotspots [6RSY, 6Q3S, 6RP9, 6RPA, 6RPB, 6VRM, 6VRN, 6VQO; light blue]. Hotspot events appear associated with a more extended R65 conformation while non-hotspot events appear associated with a kinked-back R65 conformation.

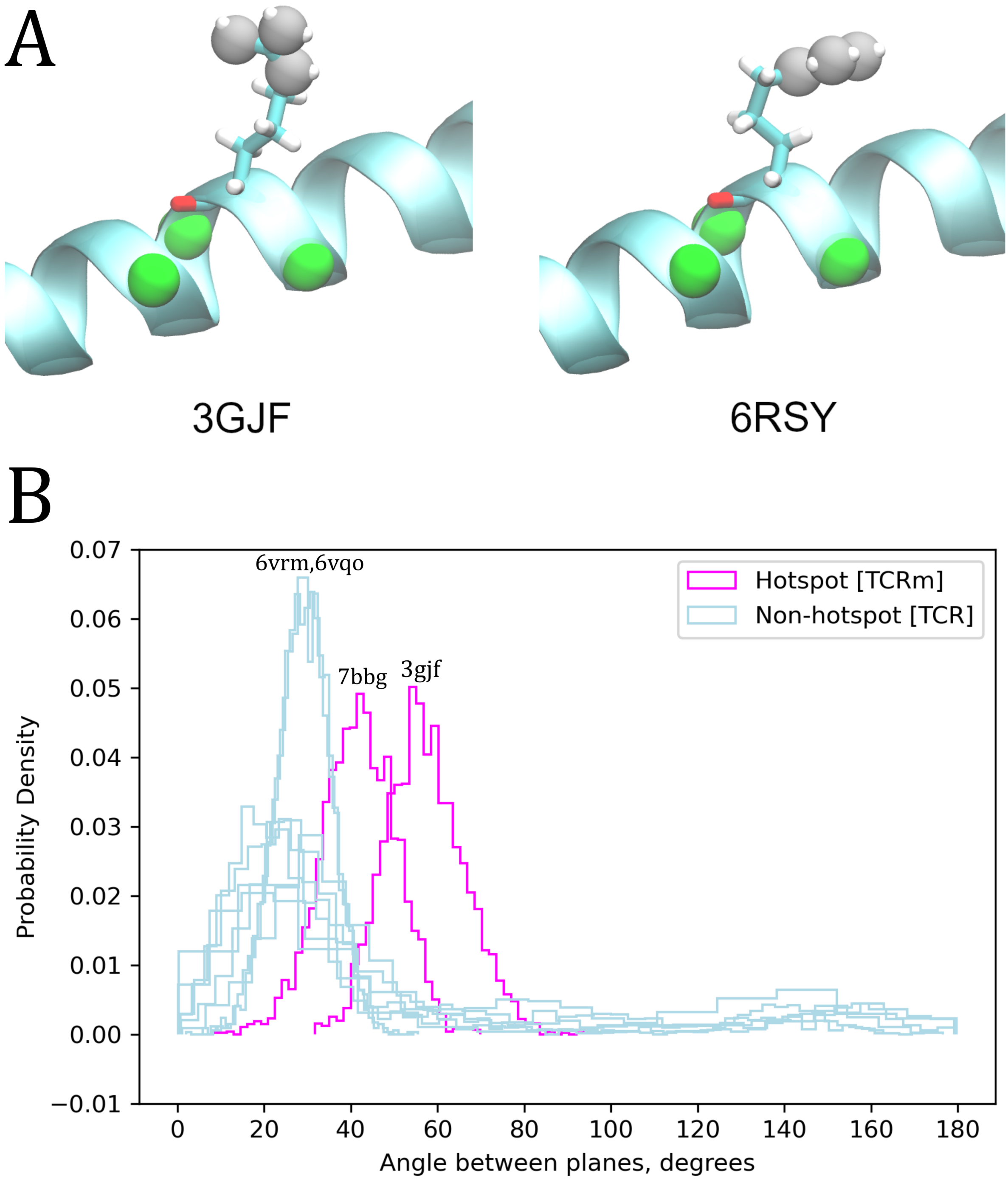

Figure S7. (A): We defined the plane of the guanidino ring using the three nitrogen atoms of the side chain (gray spheres), and the plane of the MHCα_1 helix by the Cα atoms of MHC residues 64, 66, and 68 (green spheres). This enabled the distinction between more protruding conformations (shown here for the TCRm 3M4E5, PDB structure 3GJF), and less protruding conformations (shown here for the TCR a7b2, PDB structure 6RSY). (B) R65 residues involved in hotspots (the two TCRm examples, magenta) spend more time in protruding conformations than R65 residues not involved in hotspots (the eight TCR examples, blue).

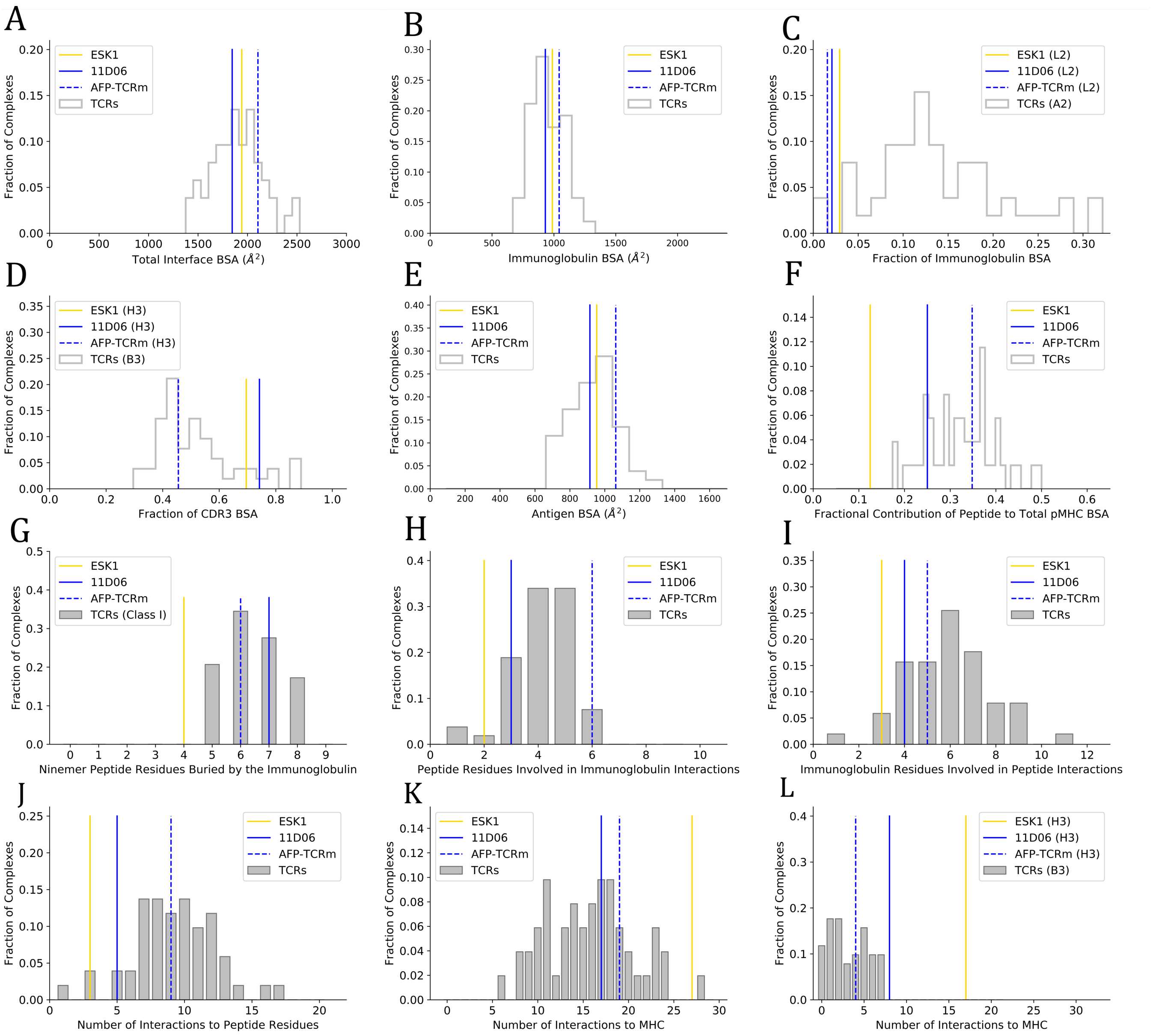

Figure S8. pMHC engagement properties of two TCRms that are currently in clinical trials (11D06 [blue solid line] and AFP-TCRm [blue dashed line]) and a TCRm suspended at the preclinical stage (ESK1 [yellow solid line]), in the context of the properties of representative TCRs [grey histograms/bars]. ESK1 and 11D06 (*i.e.* both solid lines) engage the same pMHC complex (WT1 + HLA-A*02:01). A: The total BSA across the immunoglobulin:antigen interface. B: The immunoglobulin portion of the total interface BSA. C: The fractional contribution of CDRL2 (TCRms) or CDRA2 (TCRs) to immunoglobulin BSA. D: The fractional contribution of CDRH3 (TCRms) or CDRB3 (TCRs) to CDR3 BSA. E: The pMHC portion of the total interface BSA. F: The fractional contribution of peptide BSA to pMHC BSA. G: The number of peptide residues buried in each immunoglobulin to ninemer peptide:MHC Class I complexes. H: The number of peptide residues involved in binding interactions to the TCRms/TCRs. I: The number of immunoglobulin residues involved in binding interactions to the peptide across the TCRms/TCRs. J: The number of interactions between the immunoglobulin and the peptide across the TCRms/TCRs. K: The number of interactions to MHC residues across the TCRms/TCRs. L: The number of interactions to MHC residues made by the CDRH3 (TCRms) or CDRB3 (TCRs) loop.

Interaction	Prop. in TCR:pMHC Interfaces ($\mu \pm$ sd)	Prop. in TCRm:pMHC Interfaces ($\mu \pm$ sd)
Hydrophobic	56.3% $\pm$ 10.2%	54.2% $\pm$ 8.5%
Aromatic	4.0% $\pm$ 4.3%	4.2% $\pm$ 3.9%
Polar	39.7% $\pm$ 10.7%	41.6% $\pm$ 8.0%

Table S1. The proportions of each interaction type seen across the TCR:pMHC and TCRm:pMHC interfaces. Hydrogen bonds and salt bridges are pooled as 'polar' interactions. Prop. = Proportion

Name (PDB)	CDRH1 (L)	CDRH2 (L)	CDRH3 (L)	CDRL1 (L)	CDRL2 (L)	CDRL3 (L)
Hyb3 (1W72)	GFTFDDYA (8)	ISWNSGSI (8)	ARGRGFHYYYYGMDI (15)	NIGSRS (6)	DDS (3)	QVWDSRTDHWV (11)
25-D1.16 (3CVH)	GYTFTDYN (8)	INPNNGGT (8)	ARKPYYGNFAWFAY (14)	EDIYNR (6)	GAT (3)	QQYWSTPLT (9)
3M4E5 (3GJF)	GFTFSTYQ (8)	IVSSGGST (8)	AGELLPYYGMDV (12)	SRDVGGYNY (9)	DVI (3)	WSFAGSYYV (9)
3M4F4 (3HAE)	GFTFSAYG (8)	IGSSGGGT (8)	AGELLPYYGMDV (12)	SRDVGGYNY (9)	DVI (3)	WSFAGSYYV (9)
ESK1 (4WUU)	GYSFTNFW (8)	VDPGYSYS (8)	ARVQYSGYYDWFDP (14)	SSNIGSNT (8)	SNN (3)	AAWDDSLNGWV (11)
2Q1 (6UJ9)	GFNVKYYM (8)	ISPGYDYT (8)	SRSYWRYSVDV (11)	QDVNTA (6)	SAS (3)	QQVYSSPFT (9)
H2 (6W51)	GFNVYASG (8)	IYPDSDYT (8)	SRDSSFYYVYAMDY (14)	QDVNTA (6)	SAY (3)	QQYSRYSPVT (10)
11D06 (7BBG)	GGTFSSYA (8)	IIPIFGTA (8)	ARSIELWWGGFDY (13)	QSISSW (6)	DAS (3)	QQYEDYTT (8)
3H4 (7BH8)	GYTFTDYN (8)	INPNNGGT (8)	ARPDYYGSSYGWYFDV (16)	QDINSY (6)	RAN (3)	LQYDEFPLT (9)
AFP-TCRm* (7RE7)	GYSFPNYW (8)	IDPGDSYT (8)	ARYYVSLVDI (10)	SSDVGGYNY (9)	DVN (3)	SSYTTGSRAV (10)
3.C11 (6XP6)	GGTVRSRVHA (10)	IIPIFGTA (8)	ARDVQRMGMDV (11)	QDISNW (6)	DSS (3)	QQFNSYPLT (9)

Table S2. IMGT-defined (Lefranc et al., 2003) Complementarity-Determining Region (CDR) properties of the full set of TCR-mimetic antibodies (TCRms). All bind a peptide presented by Class-I MHC, except 3.C11 which recognises a peptide presented by Class-II MHC. *AFP-TCRm refers to the unnamed clinical-stage TCRm from Liu *et al*. 2022 (Liu et al., 2022).

Antigen	Ig Name [PDB ID]	TCRm/TCR	Chain	CDRH1 Profile	CDRH2 Profile	CDRH3 Profile
WT1	ESK1 [4WUU]	TCRm	VH	GYSFTNFW	VDPGYSYS	ARVQYSG**YY**DWFDP
			VL	SSNIGS**N**T	SNN	AAWD**D**SLNGWV
	11D06 [7BBG]	TCRm	VH	GGTFSSYA	IIPIFGTA	ARSIELW**W**GGFDY
			VL	QSISSW	DAS	QQYEDYTT
	a7b2 [6RSY]	TCR*	VB	SEHNR	FQNEAQ	ASSLG**F**G**R**DVMR
			VA	TVDPNEY	GLKNN	IGGGTTSGTYKYI
NYESO-1	3M4E5 [3GJF]	TCRm	VH	GFTFSTYQ	IVSSGGST	AGELL**PY**YGMDV
			VL	SRDVGG**Y**NY	**D**VI	WSFAGSYYV
	sp3.4 [6Q3S]	TCR	VB	MNHEY	SVGAGI	ASSYVGNTGELF
			VA	DSAI**Y**N	IQSSQRE	AVRPTSGGS**Y**IPT
	NYE_S3 [6RP9]	TCR*	VB	SGHVS	FN**Y**EAQ	ASSSPGGVSTEAF
			VA	DRGSQS	IYSNGD	ALTRGPGNQFY
	NYE_S2 [6RPA]	TCR*	VB	SQVTM	ANQGSEA	SVGGSGGADTQY
			VA	VSGNPY	YITGDNLV	AVRDINSGAGS**Y**QLT
	NYE_S1 [6RPB]	TCR*	VB	MNHEY	SVGAGI	ASSY**LNR**DSALD
			VA	DRGSQS	IYSDGD	AVKSGGS**Y**IPT
p53_R175H	H2 [6W51]	TCRm	VH	GFNVYASG	IYPDSDYT	SRDSSF**Y**YV**Y**AMDY
			VL	QDVNTA	SAY	QQYSR**Y**SPVT
	12-6 [6VRM]	TCR	VB	MNHNS	SASEGT	ASSEGL**W**Q**V**GDEQY
			VA	NSASQS	VYSSG	VVQPG**G**YQKVT
	38-10 [6VRN]	TCR	VB	ENH**R**Y	SYGVKD	AISELVTGDSPLH
			VA	TSENNYY	QEAYKQQN	AFMG**Y**SGAGSYQL
	1a2 [6VQO]	TCR	VB	MNHEY	SMNVEV	ASSIQ**Q**GADTQY
			VA	NSAFQ**Y**	T**Y**SSGN	AMSG**L**KEDSSYKLI

Table S3. Decomposed per-residue energetic profiles throughout the IMGT-defined (Lefranc et al., 2003) CDRs for the case study TCR and TCRm complexes. 'Hotspots' are defined as residues predicted to have an attractive per-residue contribution of $\geq$ 7 kcal/mol to free energy based on DECOMP analysis (underlined bold red text). 'Semi-hotspots' are defined as residues that contribute between -4 and -7 kcal/mol (underlined bold black text). *Affinity-enhanced TCRs. Ig: Immunoglobulin.

Name (PDB)	Heavy V Gene (h/m, % ID)	Heavy J Gene (h/m, % ID)	Light V Gene (h/m, % ID)	Light J Gene (h/m, % ID)
Hyb3 (1W72)	IGHV3-9 (h, 97.0)	IGHJ6 (h, 92.9)	IGLV3-21 (h, 97.8)	IGLJ3 (h, 91.7)
25-D1.16 (3CVH)	IGHV1-18 (m, 95.9)	IGHJ3 (m, 100)	IGKV13-84 (m, 94.6)	IGKJ5 (m, 100)
3M4E5 (3GJF)	IGHV3-23/IGHV3D-23 (h, 92.9)	IGHJ6 (h, 100)	IGLV2-11 (h, 90.4)	IGLJ1 (h, 91.7)
3M4F4 (3HAE)	IGHV3-23/IGHV3D-23 (h, 90.8)	IGHJ6 (h, 100)	IGLV2-11 (h, 90.4)	IGLJ1 (h, 91.7)
ESK1 (4WUU)	IGHV5-10-1 (h, 87.8)	IGHJ5 (h, 100)	IGLV1-44 (h, 96.8)	IGLJ3 (h, 100)
2Q1 (6UJ9)	IGHV3-66 (h, 83.5)	IGHJ3/IGHJ4/IGHJ5/IGHJ6 (h, 85.7)	IGKV1-39/IGKV1D-39 (h, 87.0)	IGKJ1 (h, 91.7)
H2 (6W51)	IGHV3-66 (h, 83.5)	IGHJ4 (h, 92.9)	IGKV1-12/IGKV1D-13/IGKV1-16/... (h, 84.8)	IGKJ1 (h, 91.6)
11D06 (7BBG)	IGHV1-69 (h, 100)	IGHJ4 (h, 92.9)	IGKV1-5 (h, 97.8)	IGKJ1 (h, 91.7)
3H4 (7BH8)	IGHV1-18 (m, 99.0)	IGHJ1 (m, 100)	IGKV14-111 (m, 97.8)	IGKJ5 (m, 100)
AFP-TCRm* (7RE7)	IGHV5-10-1 (h, 84.7)	IGHJ3/IGHJ4/IGHJ5 (h, 85.7)	IGLV2-14 (h, 95.7)	IGLJ2/IGLJ3/IGLJ7 (h, 91.7)
3.C11 (6XP6)	IGHV1-69/IGHV1-69D (h, 96.0)	IGHJ6 (h, 100)	IGKV1-12/IGKV1D-12 (h, 91.3)	IGKJ4 (h, 91.7)

Table S4. Immunogenomic properties of the set of TCRm antibodies with solved structures at the time of this analysis. Gene identity was measured at an amino acid level by ANARCI (Dunbar and Deane, 2015); some TCRms had multiple closest genes. h: human, m: murine, ID: amino acid sequence identity. *AFP-TCRm refers to the unnamed clinical-stage TCRm from Liu *et al*. 2022 (Liu et al., 2022).

Germlines Containing **D**56 (Alleles)	Germlines Containing **E**56 (Alleles)
IGKV1-5 (01,02)	IGKV2D-26 (01,02,03)
IGKV1-13 (01,02)	IGKV2-29 (01,02,03)
IGKV1D-13 (01,02)	IGKV2D-29 (01,02)
IGKV1-33 (01)	IGKV5-2 (01,02)
IGKV1D-33 (01)	IGLV1-51 (02)
IGKV3-11 (01,02)	IGLV2-8 (01,02,03,04)
IGKV3D-11 (01,02,03)	IGLV2-14 (01,02,05)
IGKV3D-20 (01,02)	IGLV2-23 (01,02,03)
IGLV1-51 (01)	IGLV3-10 (01,03)
IGLV2-11 (01,02,03)	IGLV3-22 (01,03)
IGLV2-14 (03,04)	IGLV6-57 (01,02,03,04)
IGLV2-23 (04)	
IGLV3-21 (02,03)	
IGLV7-46 (01,02,04,05)	

Table S5. Antibody light chain germline V genes (Kappa locus in red, Lambda locus in blue) encoding a negatively-charged residue (aspartic acid [D] or glutamic acid [E]) at IMGT (Lefranc et al., 2003) position 56.

PDB ID [Ig Name]	Chains Used	Residues Rebuilt (sequential numbering)
3GJF [3M4E5]	D, E, F, K, M	None
4WUU [ESK1]	A, B, C, D, E	None
6Q3S [sp3.4]	A, B, C, D, E	None
6RP9 [NYE_S3]	A, B, C, D, E	None
6RPA [NYE_S2]	A, B, C, D, E	Chain D (145-147, 160-166, 180-184), Chain E (236-240)
6RPB [NYE_S1]	A, B, C, D, E	Chain A (221-225), Chain D (141-146)
6RSY [a7b2]	A, B, C, D, E	None
6VRM [12-6]	A, B, D, E, P	Chain D (1, 122, 125-131, 177-184)
6VRN [38-10]	A, B, D, E, P	Chain D (1, 56-61)
6VQO [1a2]	A, B, D, E, P	Chain D (1, 129-133, 182-184)
6W51 [H2]	D, E, F, O, P	None
7BBG [11D06]	A, B, C, H, L	Chain H (1, 135-141)

Table S6. Chains used in the molecular simulations and residue regions rebuilt prior to simulation. Ig: Immunoglobulin.

SUPPLEMENTARY DATASETS

Both supplementary datasets are available at https://zenodo.org/record/7220531:

Supplementary Dataset S1: Cleaned, filtered, and IMGT-numbered complexes used for the high-throughput immunoglobulin:antigen complex analysis.
Supplementary Dataset S2: PQR-format structures generated with H++ Anandakrishnan et al. (2012) for simulations of case study TCR:pMHC and TCRm:pMHC complexes.

REFERENCES

Anandakrishnan, R., Aguilar, B., and Onufriev, A. V. (2012). H++ 3.0: Automating pK prediction and the preparation of biomolecular structures for atomistic molecular modeling and simulations. *Nucleic Acids Research* 40, 537–541. doi:10.1093/nar/gks375

Berman, H. M., Westbrook, J., Feng, Z., Gilliland, G., Bhat, T. N., Weissig, H., et al. (2000). The Protein Data Bank. *Nucleic Acids Research* 28, 235–242

Brooks, B. R., III, C. L. B., A. D. Mackerell, J., Nilsson, L., Petrella, R. J., Roux, B., et al. (2009). CHARMM: The Biomolecular Simulation Program. *Journal of Computational Chemistry* 30, 1545–1614. doi:10.1002/jcc

Darden, T., York, D., and Pedersen, L. (1993). Particle mesh Ewald: An Nlog(N) method for Ewald sums in large systems. *Journal of Chemical Physics* 10089. doi:10.1063/1.464397

Dunbar, J. and Deane, C. M. (2015). ANARCI: antigen receptor numbering and receptor classification. *Bioinformatics* 32, 298–300. doi:10.1093/bioinformatics/btv552

Eastman, P., Swails, J., Chodera, J. D., McGibbon, R. T., Zhao, Y., Beauchamp, K. A., et al. (2017). OpenMM 7: Rapid development of high performance algorithms for molecular dynamics. *PLoS Computational Biology* 13, 1–17. doi:10.1371/journal.pcbi.1005659

Jorgensen, W. L., Chandrasekhar, J., and Madura, J. D. (1983). Comparison of simple potential functions for simulating liquid water. *Journal of Chemical Physics* 79. doi:10.1063/1.445869

Knapp, B., Dunbar, J., Alcala, M., and Deane, C. M. (2017). Variable Regions of Antibodies and T-Cell Receptors May Not Be Sufficient in Molecular Simulations Investigating Binding. *Journal of Chemical Theory and Computation* 13, 3097–3105. doi:10.1021/acs.jctc.7b00080

Lefranc, M.-P., Pommié, C., Ruiz, M., Giudicelli, V., Foulquier, E., Truong, L., et al. (2003). IMGT unique numbering for immunoglobulin and T cell receptor variable domains and Ig superfamily V-like domains. *Developmental and Comparative Immunology* 27, 55–77. doi:10.1016/S0145-305X(02)00039-3

Liu, C., Liu, H., Dasgupta, M., Hellman, L. M., Zhang, X., Qu, K., et al. (2022). Validation and promise of a TCR mimic antibody for cancer immunotherapy of hepatocellular carcinoma. *Scientific Reports* 12, 12068. doi:10.1038/s41598-022-15946-5

Machado, M. R. and Pantano, S. (2020). Split the Charge Difference in Two! A Rule of Thumb for Adding Proper Amounts of Ions in MD Simulations. *Journal of Chemical Theory and Computation* 16, 1367–1372. doi:10.1021/acs.jctc.9b00953

Maier, J. A., Martinez, C., Kasavajhala, K., Wickstrom, L., Hauser, K. E., and Simmerling, C. (2015). ff14SB: Improving the Accuracy of Protein Side Chain and Backbone Parameters from ff99SB. *Journal of Chemical Theory and Computation* 11, 3696–3713. doi:10.1021/acs.jctc.5b00255

Miller, B. R., Mcgee, T. D., Swails, J. M., Homeyer, N., Gohlke, H., and Roitberg, A. E. (2012). MMPBSA.py : An Efficient Program for End-State Free Energy Calculations. *Journal of Chemical Theory and Computation* 8, 3314–3321

Onufriev, A. V. and Case, D. A. (2019). Generalized Born Implicit Solvent Models for Biomolecules. *Annual Review of Biophysics* 48, 275–296. doi:10.1146/annurev-biophys-052118-115325

Salomon-Ferrer, R., Case, D. A., and Walker, R. C. (2013). An overview of the Amber biomolecular simulation package. *Wiley Interdisciplinary Reviews: Computational Molecular Science* 3, 198–210. doi:10.1002/wcms.1121

Zareie, P., Szeto, C., Farenc, C., Gunasinghe, S. D., Kolawole, E. M., Nguyen, A., et al. (2021). Canonical T cell receptor docking on peptide:MHC is essential for T cell signaling. *Science* 372, eabe9124. doi:10.1126/science.abe9124

Zhang, Z., Liu, X., Yan, K., Tuckerman, M. E., and Liu, J. (2019). Unified Efficient Thermostat Scheme for the Canonical Ensemble with Holonomic or Isokinetic Constraints via Molecular Dynamics. *Journal of Physical Chemistry A* 123, 6056–6079. doi:10.1021/acs.jpca.9b02771
